# Endovascular Coil Embolization of a Large Ruptured Splenic Artery Aneurysm: A Case Report

**DOI:** 10.7759/cureus.55054

**Published:** 2024-02-27

**Authors:** Konstantinos Roditis, Nikolaos Giannakopoulos, Vasileios Papaioannou, Konstantinos G Seretis, Theofanis Papas

**Affiliations:** 1 Department of Vascular Surgery, Korgialenio-Benakio Hellenic Red Cross Hospital, Athens, GRC

**Keywords:** endovascular coil embolization, endovascular procedures, rupture, true aneurysm, splenic artery

## Abstract

This case report aims to elucidate the current practices and efficacy of endovascular repair in managing splenic artery aneurysms (SAAs), particularly focusing on a case of a large, partially ruptured SAA. A 66-year-old female presented with severe abdominal pain and was later diagnosed with a 53mm saccular, degenerative SAA showing signs of partial rupture. The patient underwent successful endovascular repair using a combination of interlocking detachable coils and fibered coils. Despite the initial success, a follow-up CT angiogram revealed residual issues, necessitating additional embolization. The patient recovered well, with subsequent follow-ups indicating complete aneurysm closure and no complications. The successful management of this case aligns with current trends in SAA treatment, emphasizing the shift towards endovascular repair methods. This approach, highlighted in the literature, offers a minimally invasive alternative to open surgery, with lower morbidity and mortality rates. This case underscores the importance of individualized treatment planning and vigilant follow-up, particularly in light of the potential need for secondary interventions. This report contributes to the growing body of evidence supporting endovascular repair as a safe and effective treatment for SAAs, advocating for continued research into long-term outcomes and the development of advanced endovascular technologies.

## Introduction

The management of splenic artery aneurysms (SAAs) has evolved significantly over the years, with endovascular repair emerging as a preferred technique in many clinical scenarios. Splenic artery aneurysms, recognized as the third most common abdominal aneurysm, pose a significant risk of rupture, particularly in cases where they exceed 2 cm in diameter [1]. The traditional approach to SAAs has been open surgical repair, but with advancements in endovascular techniques, a shift towards less invasive methods has been observed. Endovascular repair, primarily involving coil embolization and stent grafting, offers a promising alternative with lower morbidity and mortality rates compared to open surgery [2].

A 20-year record review at Toronto General Hospital, Canada, highlighted the evolving trends in the management of SAAs, reflecting a growing preference for endovascular interventions [1]. This shift is further evidenced by successful endovascular repairs of SAAs of various sizes and complexities, as reported in multiple case studies [2,3]. For instance, a 3.1 cm SAA was effectively treated using coil embolization, demonstrating the efficacy of endovascular techniques in managing sizable aneurysms [2]. The versatility of endovascular repair is also evident in its application to complex and anomalous SAAs. Cases involving SAAs with anomalous origins or those associated with systemic conditions like lupus have been successfully managed using endovascular approaches [4,5].

This adaptability is crucial, considering the diverse presentations of SAAs. Moreover, the use of advanced technologies like multilayer stents has opened new avenues in the endovascular treatment of SAAs. These stents have been employed in complex cases, offering a minimally invasive solution while ensuring the preservation of arterial flow [6,7]. The efficacy of such innovative approaches is well-documented, with studies reporting successful outcomes and minimal complications [8].

## Case presentation

A 66-year-old woman presented to the hospital with severe abdominal pain, unaware she had a significant splenic artery aneurysm. This case was exceptional due to the aneurysm's large size and the immediate medical attention it required. There was no prior diagnosis of a splenic artery aneurysm or related conditions in her medical history. 

**Figure 1:**
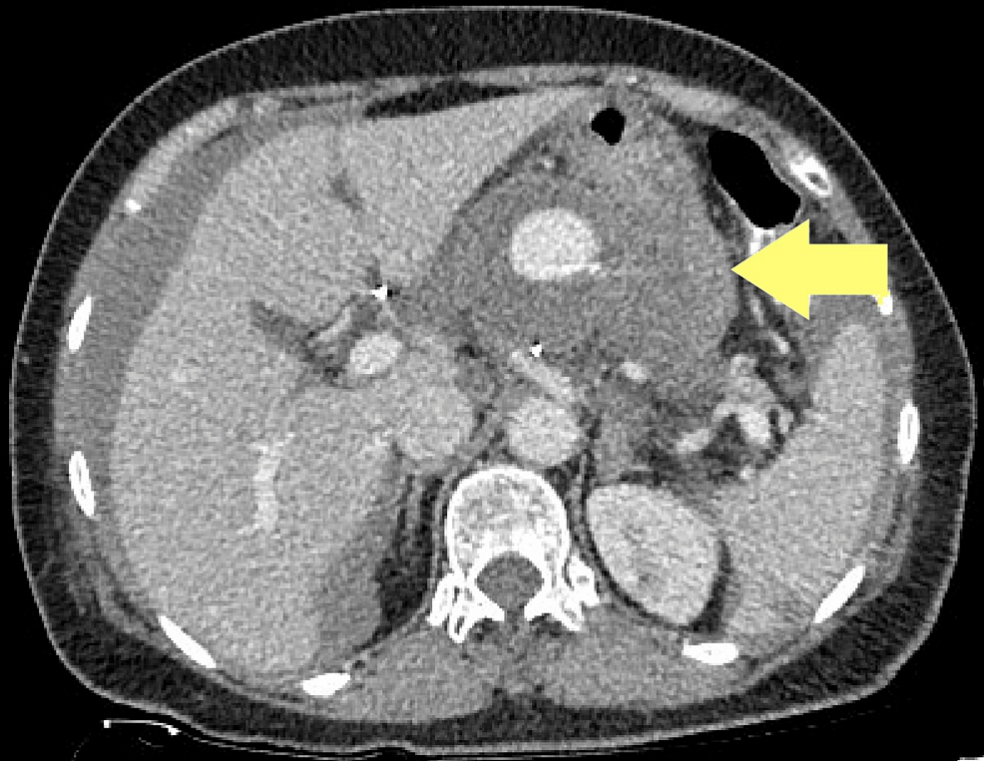
Preoperative CT angiography showing a partial rupture splenic artery aneurysm.

Diagnostic procedure with CT angiography revealed a substantial 53 mm saccular, degenerative splenic artery aneurysm with signs of partial rupture, evidenced by the presence of free fluid in the abdominal cavity. Additionally, small splenic infarcts were observed, indicating a possible thromboembolic event (Figure 1.).

**Figure 2:**
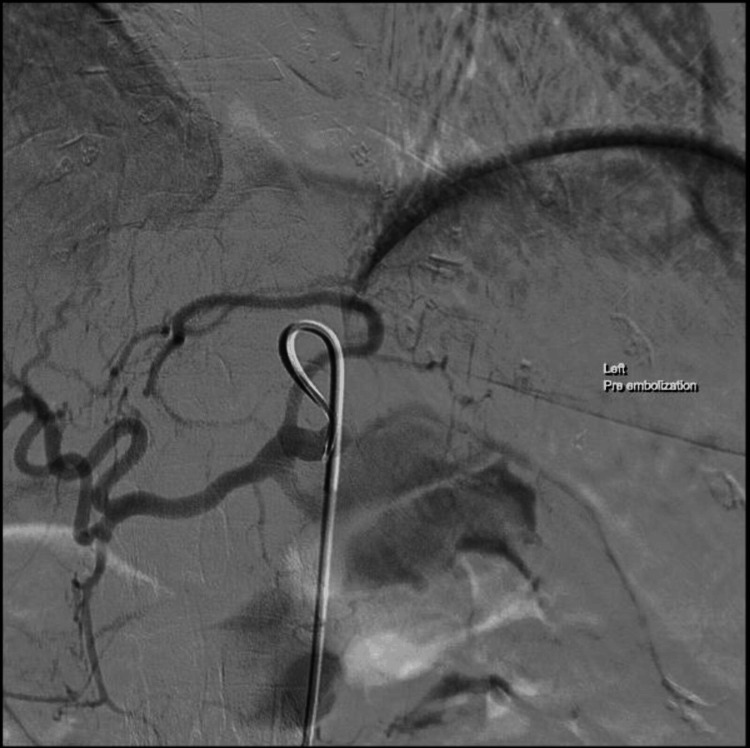
Digital subtraction angiography showing the access of ruptured splenic artery aneurysm

The patient underwent an urgent endovascular repair while in a supine position. The intervention involved a left femoral arterial approach, where a 5.1-French Elway guiding catheter (Terumo Clinical Supply Co. Ltd, Gifu, Japan), was inserted into the celiac artery through a 5.5-French sheath. A 2.8/2.2-French HyperGlide™ occlusion balloon catheter (Micro Therapeutics, Inc., Irvine, California, United States), equipped with a micro guide wire, was strategically positioned across the aneurysm's neck. The balloon was then inflated with diluted contrast material to temporarily occlude the parent artery at the aneurysm's neck (Figure 2).

**Figure 3:**
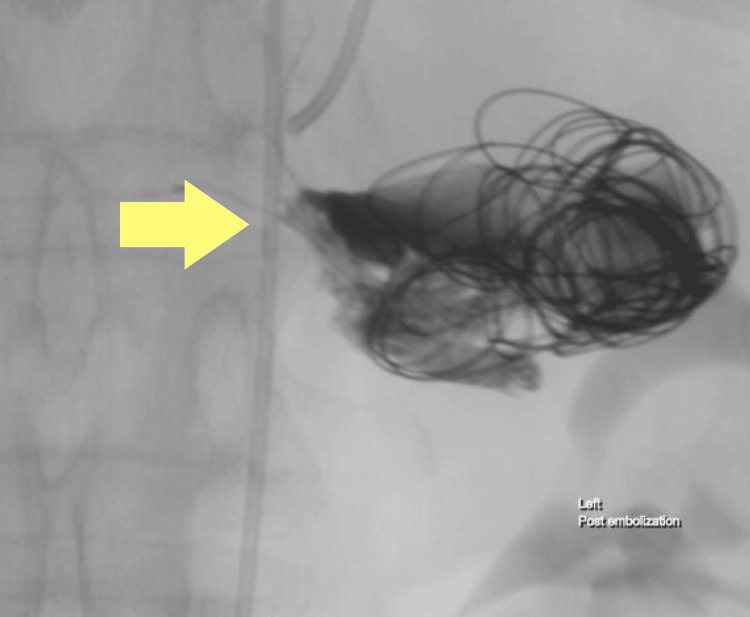
Post-embolization angiography showing complete splenic artery sealing

To secure the aneurysm, a series of nine interlocking detachable coils (IDCs) of varying sizes (two 14 mm/20 cm, three 12 mm/20 cm, two 10 mm/20 cm, two 6 mm/20 cm from Boston Scientific Corporation, Marlborough, Massachusetts, United States) and two fibered coils (Nester® embolization coils, two 8 mm/14 cm from Cook Medical, Bloomington, Indiana, United States) were deployed. After the embolization with the IDCs and fibered coils, the balloon was deflated to verify the stability of the coils within the aneurysm. Post-embolization angiography confirmed the successful closure of the aneurysm and the splenic artery's patency. The patient experienced no complications such as pain or ischemia (Figure 3).

**Figure 4:**
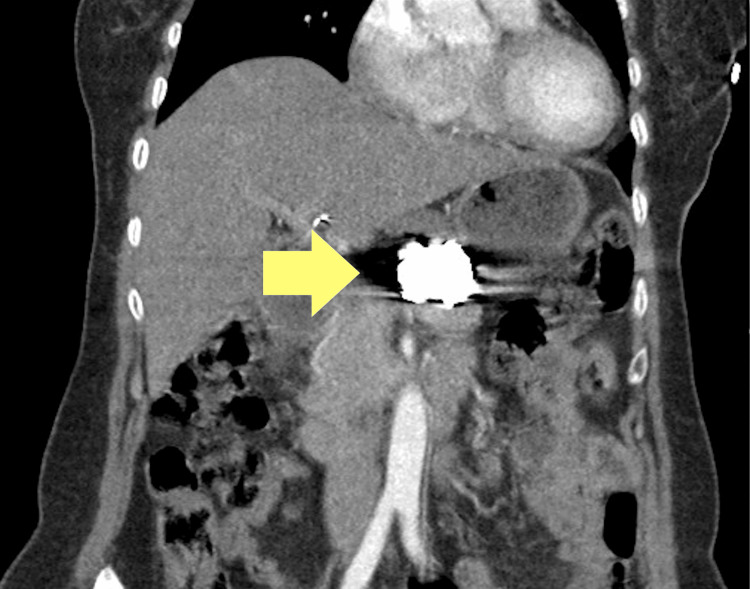
Follow-up CT angiography one year post embolisation showing complete splenic artery aneurysm sealing.

Despite the initial success of the procedure, a follow-up CT angiogram (CTA) identified some residual issues, leading to additional embolization to completely seal the aneurysm. The patient made a good recovery, she was transferred to the ward, able to move around, and was discharged on postoperative day 3 with antibiotics. At the one-year follow-up, clinical examination and CTA showed complete sealing of the aneurysm, a patent splenic artery, and no signs of splenic infarction (Figure 4). She was in good health and scheduled for follow-up in a year.

This case highlights the critical nature of rapid diagnosis and treatment in managing large splenic artery aneurysms, particularly when there is a rupture. The successful application of endovascular repair in this scenario contributes valuable knowledge to the medical field regarding the treatment of such conditions.

## Discussion

The current case of a 66-year-old woman with a large, partially ruptured SAA treated successfully with endovascular repair offers a valuable perspective when compared with existing literature. SAAs are relatively uncommon but significant, being the third most common type of abdominal aneurysm [1].

Our case aligns with the demographic trends observed in the literature, where SAAs are more frequently diagnosed in middle-aged and older individuals, particularly women [2]. The initial presentation of severe abdominal pain in our case is a common symptom of SAAs, as noted in the literature [3]. The absence of a prior diagnosis is not unusual, given that many SAAs are asymptomatic and often discovered incidentally [2]. The use of diagnostic imaging, such as CTA, in our case, is consistent with current practices and is considered the gold standard for SAA diagnosis [5].

The choice of endovascular repair in our case reflects the current trend in SAA management. Endovascular techniques, including coil embolization and stent grafting, are increasingly preferred due to their minimally invasive nature and lower associated risks compared to open surgery [1,6]. The use of coil embolization in our case aligns with common endovascular practices [7]. The technical approach, involving a guiding catheter and an occlusion balloon catheter, is in line with standard endovascular procedures. The successful outcome, marked by the absence of post-procedure complications, is consistent with the high success rates reported in the literature, where endovascular repair has shown favorable outcomes [8].

The need for additional embolization in the current case due to residual issues on follow-up CTA highlights a challenge in endovascular repair. While such secondary interventions are relatively rare, they are documented in the literature [9]. The absence of complications such as splenic infarction or ischemia in our patient is notable and desirable in endovascular repair outcomes [7]. The literature suggests that endovascular repair, including techniques used in our case, is generally effective and safe for treating SAAs. The growing body of evidence supports its use as a first-line treatment option, especially in cases where open surgery poses higher risks [1,6]. However, endovascular repair is not without its challenges. Cases of failed endovascular repair leading to complications like the Kasabach-Merritt phenomenon have been reported, underscoring the need for careful patient selection and technique refinement [8].

Despite these challenges, the overall safety and effectiveness of endovascular stent grafting in preserving blood flow and preventing splenic infarction have been recognized, with some studies suggesting its superiority over traditional coil embolization [9]. In conclusion, the landscape of SAA management is continually evolving, with endovascular repair standing out as a safe, effective, and versatile option. 

## Conclusions

Our case reinforces the efficacy and safety of endovascular repair for SAAs, highlighting the importance of individualized treatment and vigilant follow-up, particularly considering the potential need for secondary interventions. The successful outcome aligns with current trends and supports the increasing reliance on endovascular techniques in SAA management. However, this report has its limitations. The findings are based on a single patient's experience, which limits the generalizability of the results. Additionally, the long-term outcomes beyond the one-year follow-up period have not been explored. Future research should focus on the long-term outcomes of endovascular repair for SAAs, with an emphasis on evaluating the durability of the treatment over extended periods. There is also a need to investigate emerging endovascular technologies and techniques that could further enhance treatment efficacy and minimize the necessity for secondary interventions. This case contributes to the growing body of evidence, advocating for the continued evolution and refinement of endovascular approaches in the management of SAAs
